# ChatGPT-4o in delirium recognition: a pilot study

**DOI:** 10.1590/1806-9282.20260268

**Published:** 2026-08-10

**Authors:** Kubra Cingar Alpay, Suna Avci, Aysegul Gunduz, Duygu Ozata, Gokalp Kurthan Avlagı, Seyda Bilgin, Ummugulsum Durak, Alper Doventas, Deniz Suna Erdincler

**Affiliations:** 1 Istanbul University-Cerrahpasa, Cerrahpasa Medical School, Department of Internal Medicine, Division of Geriatrics – Istanbul, Turkey.; 2 Istanbul University-Cerrahpasa, Cerrahpasa Medical School, Department of Neurology – Istanbul, Turkey.

**Keywords:** Delirium, Geriatrics, Artificial intelligence, Large language models

## Abstract

**OBJECTIVE:**

Delirium is common yet frequently under-recognized in older adults and is associated with increased morbidity and mortality. Large language models may support clinical reasoning, but their performance and consistency in complex geriatric syndromes remain unclear.

**METHODS:**

We analyzed 20 PubMed-indexed geriatric case reports converted into structured vignettes with explicit references to delirium removed. ChatGPT-4o was tested in three conditions (two independent sessions and one consecutive-session run) to assess delirium identification and temporal consistency; DeepSeek-V2 served as a comparator. Two clinicians (geriatrician and neurologist) independently rated outputs for clinical plausibility, concordance with the reference diagnosis, and decision-support value.

**RESULTS:**

ChatGPT-4o identified delirium in 70% of cases in both independent sessions and 90% in the consecutive-session run, whereas DeepSeek-V2 identified 50%. Differences across ChatGPT-4o conditions were not significant (p=0.135). Agreement between independent ChatGPT-4o sessions was substantial (κ=0.76), while agreement involving the consecutive-session run was low, suggesting contextual priming. Expert ratings were higher in successfully identified cases across domains (all p<0.05). Inter-rater reliability was highest for concordance (Intraclass Correlation Coefficient=0.86).

**CONCLUSION:**

ChatGPT-4o showed promising but context-sensitive delirium recognition in geriatric vignettes. Large language models may serve as vigilance-enhancing decision-support tools under expert supervision, pending prospective validation.

## INTRODUCTION

Delirium is an acute, fluctuating neurocognitive syndrome with disturbances in attention and awareness^1^. It affects 14.7% of hospitalized older adults and up to 80% of mechanically ventilated patients^2,3^. Delirium is associated with more than a twofold increase in 30-day mortality, even after adjustment for comorbidities^2^. Despite its impact, delirium is frequently missed due to heterogeneous presentations, overlap with dementia, and limited routine cognitive screening^1^.

In older adults, multimorbidity, polypharmacy, and age-related vulnerability increase delirium risk^1,4^. Hypoactive delirium is especially difficult to recognize because it can resemble depression or baseline cognitive impairment^5,6^.

These challenges support the need for tools that assist systematic delirium recognition in high-risk populations. Large language models (LLMs) are increasingly being explored for their potential to process complex clinical data^7^. Recent work suggests LLMs can approach clinician-level diagnostic performance in structured scenarios^8^. Initial studies evaluating the diagnostic and decision-support capabilities of LLMs in geriatrics have shown promising but still preliminary results. In one study, ChatGPT-4 demonstrated the ability to administer and interpret structured screening tools such as the Sour Seven Questionnaire for delirium detection, showing performance comparable to that of human clinicians^7^. Similarly, LLMs have been tested in polypharmacy scenarios using Beers and Screening Tool of Older Persons’ Prescriptions criteria, though expert oversight remained essential for final decisions^9^.

Limitations include hallucinated outputs and reduced reliability with incomplete or ambiguous inputs^10^.

This pilot study evaluates ChatGPT-4o's ability to identify delirium in blinded geriatric clinical vignettes, compared with expert assessments. We also examine cross-session consistency to explore temporal reproducibility. The goal is to provide preliminary evidence for LLMs as vigilance-enhancing tools under clinician supervision.

## METHODS

### Study design and case selection

We conducted a pilot case series using PubMed-indexed case reports involving older adults. The search was performed in July 2025 using the keywords "delirium," "older adults," and "geriatric," limited to the previous 5 years. Two clinicians screened 51 records and included reports in which delirium was the final diagnosis and sufficient clinical detail was available to construct a blinded vignette. Cases with incidental mention of delirium or insufficient data were excluded. After screening, 20 cases were retained for analysis. No non-delirium controls were included, consistent with the case-only feasibility design.

### Case preparation and blinding

For each selected case, a structured clinical vignette was prepared. Cases were converted into structured vignettes, and explicit mentions of "delirium" were removed to ensure blinded model assessment.

### Large language model evaluations (ChatGPT-4o and DeepSeek)

Clinical vignettes were submitted to ChatGPT-4o (OpenAI) and DeepSeek-V2 (DeepSeek) in July 2025 using a standardized prompt: "*Based on the clinical information above, provide three differential diagnoses in order of relevance, followed by a single most likely final diagnosis. Justify each briefly.*"

ChatGPT-4o was tested in two independent sessions (new chat in each case) and one consecutive-session run (all cases in one thread) to explore context effects; DeepSeek-V2 was evaluated once as a comparator.

### Outcome measures and terminology

**Figure 1:**
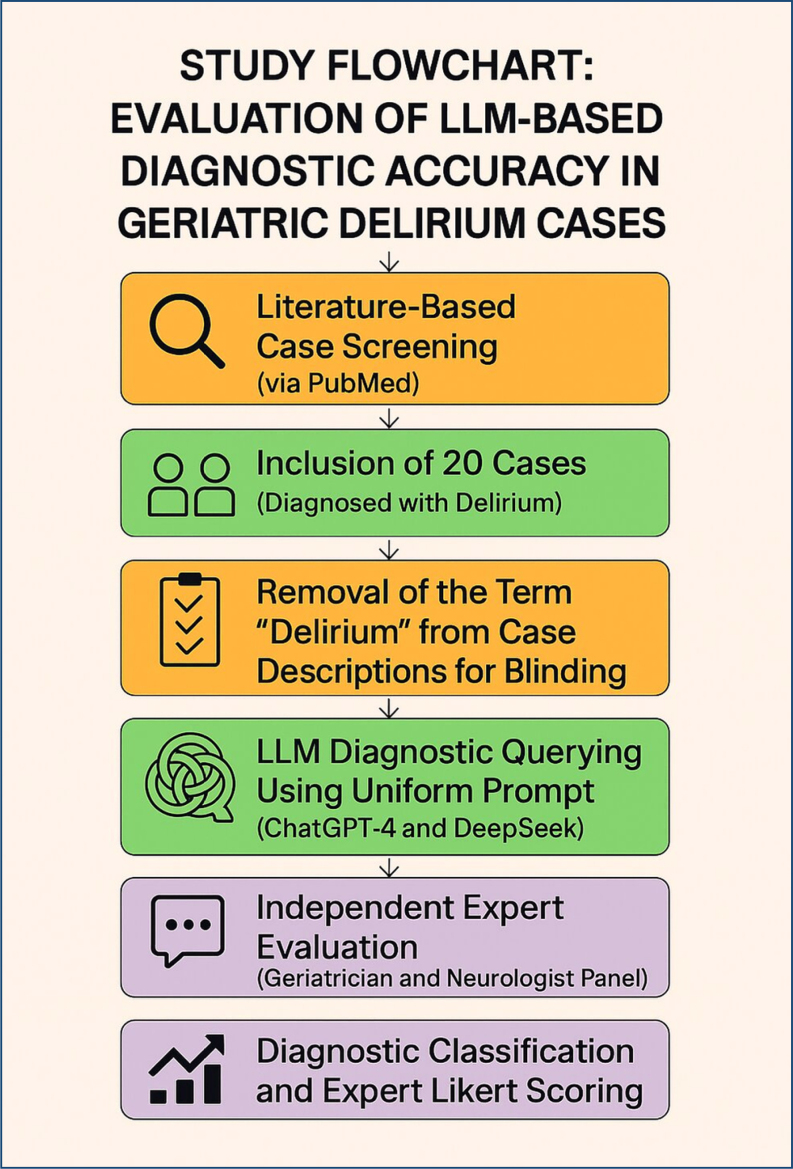
Study flowchart for large language model-based diagnostic evaluation in geriatric delirium.

Because the study was case-only, outcomes were defined as identification-based. Delirium was considered "identified" if it appeared in the top-3 differential diagnoses; otherwise, it was classified as "missed." Only the explicit term "delirium" was accepted. The primary outcome was the identification rate; secondary outcomes were top-1 ranking and final diagnosis selection. The study flow is shown in Figure 1.

### Expert evaluation

All model outputs were independently reviewed by two clinical experts (a geriatrician and a neurologist), each with over ten years of clinical experience. Each suggested diagnosis was rated using a five-point Likert scale under the following predefined dimensions:

Clinical plausibility—whether the diagnosis was reasonable given the case details,

Concordance with reference diagnosis—whether the diagnosis reflected the correct underlying condition,

Decision support value—whether the output could assist a clinician in reaching the correct conclusion.

### Statistical analysis

Identification rates are presented as proportions with 95%CI (Clopper-Pearson). Likert ratings are summarized using medians (IQR) and compared between identified vs. missed cases using the Mann-Whitney U test. Differences in identification rates across ChatGPT-4o conditions were assessed using Cochran's Q test with McNemar post-hoc tests (Bonferroni-adjusted). Agreement in identification across conditions was evaluated with Cohen's kappa. Inter-rater reliability for Likert ratings was assessed using Intraclass Correlation Coefficient (ICC) (two-way random-effects, absolute agreement). Analyses were performed in Statistical Package for the Social Sciences v27; p<0.05 was considered significant.

### Ethical considerations

The study used publicly available case reports without identifiable data; ethics approval was not required.

## RESULTS

Diagnostic identification rates and confidence intervals ChatGPT-4o identified delirium in 70% of cases in both independent sessions (14/20; 95%CI 45.7–88.1). In the consecutive-session condition, identification increased to 90% (18/20; 95%CI 68.3–98.8). DeepSeek-V2 identified delirium in 50% of cases (10/20; 95%CI 27.2–72.8).

### Comparison of evaluation conditions

**Figure 2:**
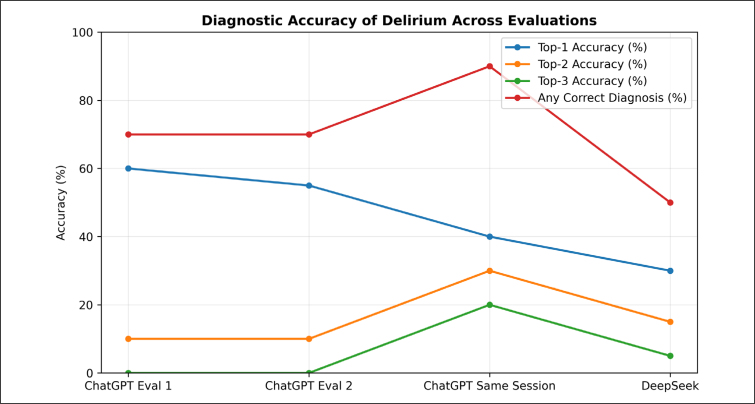
Rank position of delirium identification across ChatGPT-4o session conditions and DeepSeek-V2.

Identification rates did not differ significantly across ChatGPT-4o conditions [Cochran's Q(2)=4.00, p=0.135]. Pairwise McNemar tests showed no significant differences between runs (all p>0.05). The consecutive-session condition showed a higher identification rate than DeepSeek-V2 (90 vs. 50%; p=0.021), though this finding should be interpreted cautiously given multiple comparisons. The increased performance in the consecutive session is consistent with possible contextual carry-over effects (Figure 2).

### Intra-model consistency (reliability)

Agreement between independent sessions was substantial (κ=0.762). In contrast, agreement between independent and consecutive-session conditions was low (Run 1 vs. Run 3: κ=0.118; Run 2 vs. Run 3: κ=-0.176), indicating context-dependent variability across evaluation settings.

### Expert evaluation and clinical relevance

Expert ratings were higher in successfully identified cases across all domains.

Concordance with the reference diagnosis was significantly greater (median 5.0 vs. 1.5; p<0.001).

Decision-support value was also higher (median 4.5 vs. 3.25; p<0.001).

Clinical plausibility showed a smaller but significant difference (median 4.5 vs. 3.25; p=0.033).

### Inter-rater reliability

Inter-rater reliability was highest for concordance (ICC=0.863), followed by decision-support value (ICC=0.419) and clinical plausibility (ICC=0.228).

### Case-by-case diagnostic suggestions

Table 1 summarizes, for each case vignette, the three differential diagnoses and the single most likely final diagnosis generated by ChatGPT-4o.

**Table 1:** Case-level differential diagnoses and final diagnosis generated by ChatGPT-4o.

Case No	Top 3 differential diagnoses	Final diagnosis
1	Delirium superimposed on dementia; Lewy body dementia; drug side effect	Delirium superimposed on dementia
2	Sepsis-associated encephalopathy; hepatic encephalopathy; encephalitis	Sepsis-associated encephalopathy
3	Delirium due to urosepsis; obstructive pyelonephritis; end-stage dementia	Delirium due to urosepsis
4	Hypercalcemic encephalopathy; delirium secondary to hypercalcemia; drug-induced neuropsychiatric syndrome	Hypercalcemic encephalopathy
5	Metabolic delirium; psychotic major depression; delirium superimposed on MCI	Metabolic delirium
6	Parkinson's disease, dementia; vascular dementia; polypharmacy and drug interaction	Parkinson's dementia
7	Delirium tremens (alcohol withdrawal); acute renal failure; alcoholic liver disease and hypoglycemia	Delirium tremens
8	Cervical abscess and systemic infection; cerebrovascular event; sepsis	Retropharyngeal abscess-induced systemic infection
9	Delirium superimposed on dementia; colonic dilatation; urinary tract infection	Delirium superimposed on dementia
10	Immune checkpoint inhibitor-induced autoimmune encephalopathy/limbic encephalitis; immune-related hyperthyroidism/thyrotoxicosis; paraneoplastic limbic encephalitis	Immune checkpoint inhibitor-induced autoimmune encephalopathy
11	Postoperative delirium; drug toxicity; hypoglycemia/metabolic encephalopathy	Postoperative delirium
12	PPI-induced hypomagnesemia-related encephalopathy; hypocalcemia-related encephalopathy; CNS sarcoidosis	PPI-related encephalopathy
13	Delirium triggered by clozapine; clozapine toxicity/sensitivity; encephalopathy due to AChEI and clozapine interaction	Delirium triggered by clozapine
14	Intraoperative onset of delirium; antipsychotic side effect/drug interaction; psychotic decompensation in schizophrenia	Intraoperative onset of delirium
15	Post-ICU delirium; hypoxic-ischemic encephalopathy; post-stroke vascular cognitive impairment	Post-ICU delirium
16	Postoperative delirium; sedative-related iatrogenic agitation; delirium related to environmental isolation	Postoperative hyperactive delirium
17	Acute sepsis-related delirium; metabolic encephalopathy (Na, Mg deficiency); postoperative delirium	Acute sepsis-related delirium
18	Pulmonary embolism and hypoxic delirium; hypoxic/hypoperfusion encephalopathy; delirium due to acute pneumonia and infection	Pulmonary embolism and hypoxic delirium
19	Neuroborreliosis; psychosis/delirium complicated by dementia; paraneoplastic or inflammatory encephalopathy	Neuroborreliosis (Lyme disease)
20	Lithium toxicity; acute renal failure encephalopathy; dehydration and electrolyte imbalance	Lithium toxicity

ICU: Intensive Care Unit; CNS: Central Nervous System; PPI: proton pump inhibitor; MCI: Mild Cognitive Impairment; AChEI: Acetylcholinesterase Inhibitor.

## DISCUSSION

Across 20 cases, ChatGPT-4o demonstrated higher delirium identification rates than DeepSeek, identifying delirium in 70% of cases across independent sessions and 90% in the consecutive-session condition. The lower identification rate observed with DeepSeek-V2 should be interpreted cautiously, as the between-model comparison was not fully symmetrical. Differences in model behavior, prompting context, or training characteristics may also have contributed to the observed variation^11^. The higher identification rate observed in the consecutive-session condition likely reflects contextual carry-over effects, whereby prior case exposure influences subsequent outputs through persistent conversational context^12^. In contrast, the consistent 70% identification rate across independent sessions may better represent baseline performance. The 30% false-negative rate, however, underscores the clinical risk of missed diagnoses and reinforces the necessity of physician oversight in acute care settings.

Expert evaluations confirmed that successful identification was associated with significantly higher ratings for concordance, decision-support value, and clinical plausibility. These findings highlight that diagnostic utility extends beyond numerical identification rates and requires qualitative clinical validation^13^. Inter-rater agreement was highest for concordance with the reference diagnosis, whereas agreement was lower for clinical plausibility and decision-support value. This pattern suggests that recognizing whether an output broadly aligns with the correct syndrome may be more reproducible than judging how clinically convincing or practically useful that output is. In other words, apparent diagnostic agreement does not necessarily translate into uniform interpretation of the model's qualitative reasoning. Prior delirium methodology research has also highlighted variability in reference-rater practices and emphasized the importance of inter-rater reliability and standardization^14^. This variability may also partly reflect differences in disciplinary emphasis between geriatrics and neurology.

Our findings align with previous work exploring LLM applications in geriatric contexts. Choi et al. demonstrated that structured prompt adaptation improved ChatGPT performance in administering the Sour Seven delirium screening tool^7^. However, their study did not assess temporal reproducibility or repeated evaluations. In contrast, our investigation examined model consistency across sessions without additional fine-tuning. Similarly, Socrates et al. reported that GPT-4o performed well in structured deprescribing frameworks but lost accuracy in complex reasoning stages^9^, paralleling the missed identifications observed in clinically intricate delirium cases in our cohort. Beyond clinician-facing decision support, LLMs may also contribute to patient-centered tasks in geriatrics; for example, Artificial Intelligence (AI)-assisted Percutaneous Endoscopic Gastrostomy (PEG) aftercare education has been shown to be feasible, although clinician refinement remains necessary to ensure clinical appropriateness^15^.

Performance variability across medical domains has also been reported. In endodontic education, ChatGPT-4o showed limited reliability and weak agreement with expert scoring^16^, suggesting that LLM performance remains task- and context-dependent. This reinforces that apparent "expert-level" outputs must be interpreted cautiously, particularly in complex geriatric syndromes.

Several limitations merit consideration. The small sample size and exclusive reliance on published case reports may limit generalizability and introduce publication bias. Prompt phrasing was not varied, and alternative formulations could influence outputs. In addition, the comparison between ChatGPT-4o and DeepSeek-V2 should be interpreted cautiously because the evaluation process was not fully symmetrical: ChatGPT-4o was assessed across three session conditions, whereas DeepSeek-V2 was evaluated once as a comparator. Furthermore, newer versions of both model families became available after the study period and were not examined in the present analysis. Given the rapid evolution of large language models, these findings should therefore be considered model-, version-, and time-specific. Another important limitation is the absence of a non-delirium control group. Because only cases with delirium as the reference diagnosis were included, the present design supports an identification-based assessment but does not allow estimation of specificity, false-positive rates, or discrimination against competing conditions such as dementia progression, primary psychiatric disorders, or non-delirious encephalopathy. Accordingly, these findings should not be interpreted as a complete measure of diagnostic accuracy in real-world clinical settings. Consistent with known LLM constraints, the model remains vulnerable to hallucinated reasoning, reduced performance with incomplete data, and semantic substitutions—such as misclassifying delirium as encephalopathy—likely driven by overlapping symptomatology^17,18^.

AI-assisted decision-making in geriatrics should be framed as augmentation rather than automation. Our findings support positioning ChatGPT-4o as a vigilance-enhancing tool within supervised, interdisciplinary workflows rather than as an autonomous diagnostic agent^19,20^.

## CONCLUSION

This pilot case series suggests that ChatGPT-4o can identify delirium patterns in structured geriatric vignettes, with stable performance across independent sessions but context-sensitive variation in sequential testing. Although identification rates were promising, the persistent false-negative rate and variability in expert appraisal underscore the need for specialist oversight. Large language models may serve as supplementary decision-support tools in geriatric care, provided they are implemented within structured, interdisciplinary clinical frameworks and validated in prospective real-world settings.

## Data Availability

The datasets generated and/or analyzed during the current study are available from the corresponding author upon reasonable request.
